# A new species of *Phalacrostemma* (Annelida, Sabellariidae) from the West Mariana Ridge, northwestern Pacific

**DOI:** 10.3897/zookeys.1282.191792

**Published:** 2026-06-17

**Authors:** Eijiroh Nishi, Genki Kobayashi, Naoto Jimi, Yoshihiro Fujiwara, Elena K. Kupriyanova

**Affiliations:** 1 College of Education, Yokohama National University, Hodogaya, Yokohama 240-8501, Japan Australian Museum Research Institute, Australian Museum Sydney Australia; 2 The Center for Molecular Biodiversity Research, National Museum of Nature and Science, 4-1-1 Amakubo, Tsukuba, Ibaraki 305-0005, Japan College of Education, Yokohama National University Yokohama Japan; 3 Environment and Safety Sciences Course, Department of Educational Collaboration, Osaka Kyoiku University, 4-698-1 Asahigaoka, Kashiwara, Osaka 582-8582, Japan Environment and Safety Sciences Course, Department of Educational Collaboration, Osaka Kyoiku University Kashiwara Japan; 4 Sugashima Marine Biological Laboratory, Graduate School of Science, Nagoya University, 429-63 Sugashima, Toba, Mie 517-0004, Japan The Center for Molecular Biodiversity Research, National Museum of Nature and Science Tsukuba Japan; 5 Centre for Marine and Coastal Studies, Universiti Sains Malaysia 11800 USM, Penang, Malaysia Sugashima Marine Biological Laboratory, Graduate School of Science, Nagoya University Sugashima Japan; 6 Research Institute for Marine Technology and Engineering, Japan Agency for Marine-Earth Science and Technology (JAMSTEC), 2–15 Natsushima-cho, Yokosuka 237–0061, Japan Centre for Marine and Coastal Studies, Universiti Sains Malaysia Penang Malaysia; 7 Australian Museum Research Institute, Australian Museum, 1 William Street, Sydney 2010 NSW, Australia Research Institute for Marine Technology and Engineering, Japan Agency for Marine-Earth Science and Technology (JAMSTEC) Yokosuka Japan; 8 Department of Biological Sciences, Macquarie University, North Ryde NSW 2109, Australia Department of Biological Sciences, Macquarie University North Ryde Australia

**Keywords:** Deep sea, Offshore Seabed Natural Environment Conservation Area, polychaete, Ritto Seamount, taxonomy

## Abstract

A new species of *Phalacrostemma* (Annelida, Sabellariidae) is described based on the specimens collected at a depth of 675 m on the Ritto Seamount of the West Mariana Ridge, northwestern Pacific. This record represents both the deepest occurrence of Sabellariidae in Japan and the first record of the genus *Phalacrostemma* from the region. *Phalacrostemma ritto***sp. nov**. dwells in a tube made of small sand particles and foraminiferans, which is attached to the spines of a cidarid sea urchin. The new species is characterized by having 22–30 pairs of outer paleae arranged in a spiral, outer paleae with acute tips and compact thecae, one or two inner paleae in each row, 8–13 opercular papillae on each side, five pairs of nuchal hooks with curved tips, a pair of slender ventral lobes on the first chaetiger, a single pair of conical papillae on the second chaetiger, bilobed notopodia in the middle to posterior abdomen, and abdominal uncini bearing three rows of teeth. A phylogenetic analysis, which included the new species and the newly sequenced *Idanthyrsus okudai* and *Sabellaria isumiensis* and was based on sequences of four gene fragments (COI, 16S, 18S, and 28S), was conducted. The new species was nested within the fully supported *Phalacrostemma* clade and recovered as the sister taxon to *Gesaia csiro*, whereas support values for phylogenetic relationships among sabellariid species were generally low.

## Introduction

The family Sabellariidae Johnston, 1865 comprises 150 species in 12 genera (Read and Fauchald 2025a). Most sabellariids inhabit intertidal to sublittoral zones, although the genera *Mariansabellaria*, *Phalacrostemma*, *Gesaia*, *Bathysabellaria*, and *Tetreres* are known exclusively from the deep-sea environments (Capa and Hutchings 2014). The deep-sea genus *Phalacrostemma* Marenzeller, 1895 currently includes 15 valid species (Read and Fauchald 2025b). The shallowest record is for *P. gloriaae* Kirtley, 1994, which was reported from the Gulf of Mexico at a depth of 228 m (Zhang et al. 2020), whereas the deepest records are for *P. perkinsi* Kirtley, 1994 from the Bahamas at 3000 m, and an unidentified *Phalacrostemma* species from the North Atlantic at the same depth (Chávez-López 2022).

A notable morphological feature of *Phalacrostemma* is its tube, which is composed of sand granules and dead foraminiferans (Kirtley 1994; Lechapt and Kirtley 1998; Chávez-López 2022). Some species attach their tubes to hard substrates, such as molluscan shells and sea urchin spines. For example, *P. maloga* Hutchings, Capa & Peart, 2012 was reported from a molluscan shell (Hutchings et al. 2012), *P. timoharai* Zhang, Hutchings, Burghardt & Kupriyanova, 2020 was living on a molluscan shell as a solitary individual (Zhang et al. 2020), *P. cidariophilum* Marenzeller, 1895 was reported from sea urchin spines, and *P. dorothyae* Kirtley, 1994 was found attached to both a gastropod shell and sea urchin spines (Chávez-López 2022).

In Japanese waters, five genera and 14 species of Sabellariidae have been recorded (Nishi and Kato 2002; Nishi et al. 2010; Jimi 2024; Nishi et al. 2025), and there are currently no confirmed records of *Phalacrostemma*. *Phalacrostemma elegans* Fauvel, 1911, reported by Imajima (2006) from 78–79 m off the Miura Peninsula in Sagami Bay without a description of its morphological characters, was subsequently transferred to the genus *Gesaia* by Kirtley (1994). Furthermore, records of deep-sea sabellariids other than *Phalacrostemma* from waters around Japan remain scarce; only *Lygdamis giardi* (McIntosh, 1885) has been reported from depths of 40–250 m in Tosa Bay (Imajima 2001) and 422–425 m off the Pacific coast of northern Honshu (Imajima 2009).

**Figure 1.**
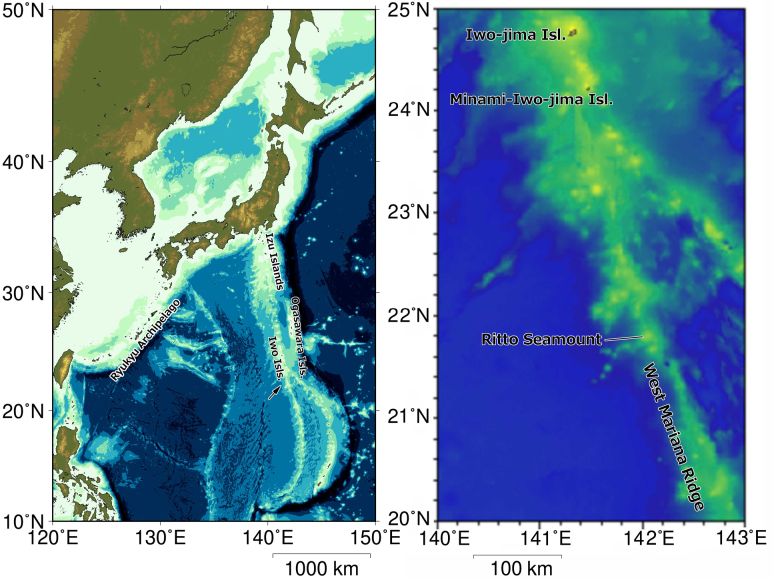
Map of the collection site in Japanese waters (left) and the Western Mariana Ridge, Ritto Seamount, Japan (right). The arrow on the left indicates the location of Ritto Seamount and the adjacent area. The base map on the right has been modified from the U.S. NOAA SRTM15_PLUS dataset.

Offshore Seabed Nature Conservation Areas are deep-sea marine protected zones (MPA) designated in December 2020 under Japan’s Nature Conservation Act (Ministry of the Environment 2020a, 2020b). These areas include four regions: the southernmost part of the Japan Trench and areas around the Izu-Ogasawara Trench; areas including the Naka-Mariana Ridge and West Mariana Ridge; areas including the Nishi-Shichito Ridge; and the northern part of the Mariana Trench. Five research voyages onboard the research vessel (RV) *Kaimei* in 2020–2022 and the deep-sea submersible support vessel *Yokosuka* in 2022–2024 documented unique deep-sea faunae, including new and rarely recorded species. Ritto Seamount, located in the southern part of the Offshore Seabed Natural Environment Conservation Area (Fig. 1), has yielded three new species of decapod crustaceans (Komai et al. 2023a, 2023b), as well as rare decapods and newly recorded fishes (Komai et al. 2022; Koeda et al. 2024). However, no annelids have yet been recorded from the sothern part of the area.

In the present study, we describe a new species of *Phalacrostemma* collected from the bathyal zone of Ritto Seamount. This finding represents the deepest record of Sabellariidae in Japanese waters and the first confirmed record of *Phalacrostemma* from Japan. The individuals inhabit tubes composed of small sand particles and foraminiferans attached to sea urchin spines.

## Materials and methods

**Figure 2.**
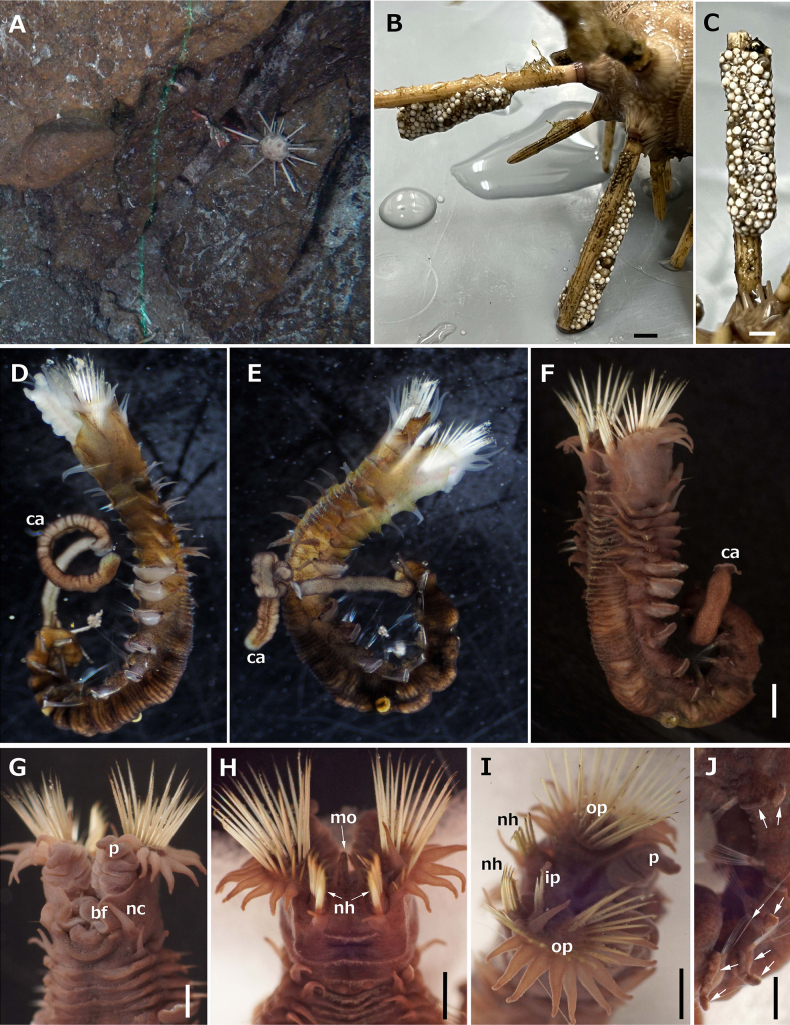
*Phalacrostemma ritto* sp. nov. **A**. Habitat of the sea urchin of the family Cidaridae; **B**. Tubes attached to sea urchin spines; **C**. Close-up view of the tube; **D, E**. Live animal (holotype); **F**. Dorso-lateral view of preserved specimen (holotype); **G**. Ventral view of head and operculum; **H**. Dorsal view of head and operculum; **I**. Anterior view of operculum; **J**. Middle to posterior abdomen, arrows indicate lobes of notopodia. Abbreviations: bf: buccal flap; ca: cauda; ip: inner palea; mo: median organ: nc: neuropodial cirri; nh: nuchal hook; op: outer palea; p: palp. Scale bars: 0.5 mm.

Benthic samples were collected during a dive of the deep-sea submergence vehicle (DSV) *Shinkai 6500* operated from RV*Yokosuka*, during research cruise YK24-15C at Ritto Seamount, West Mariana Ridge, in October 2024 (Fig. 1). An unidentified cidarid sea urchin collected during this cruise bore short polychaete tubes composed of sand grains and dead foraminiferans attached to its spines (Fig. 2A–C). Two worms were extracted from the tubes, and photographs of the living individuals were taken on board (Fig. 2D, E). The worms and their tubes were fixed and preserved in 70% ethanol.

Digital photographs of the preserved specimens and tubes were taken using a Sony α77D camera equipped with a Minolta Macro-Zoom 1–3× lens. Photographs of chaetae were taken with an iPhone 7 (Apple Inc.) connected to a Leica CM-E compound microscope. The iPhone 7 camera was inserted into the eyepiece tube of the compound microscope through the microscope adapter i-NTER LENS, and optimal images were captured using Micronet i-NTER SHOT software. The microscope adapter and associated application software were manufactured by MeCan Imaging Co. Ltd, Saitama, Japan. Digital images were edited using ®︎Adobe Photoshop Elements version 10 (Adobe Systems Inc.).

For scanning electron microscopy (SEM), the parapodia with chaetal tufts were dehydrated through a gradual series of ethanol for 10 min each and finally washed with 100% ethanol for 10 min. The samples were then washed with a 1:1 and 1.5:0.5 mixture of 100% ethanol and hexamethyldisilazane (HMDS) for 10 min each, followed by 100% HMDS for 10 min as per Nation (1983) and Nishi et al. (2022a). Specimens were left overnight to ensure HMDS evaporation, then sputter-coated with platinum and examined using a Hitachi FE SU8010 at the Instrumental Analysis Center of Yokohama National University.

Terminology for morphological description follows Capa and Hutchings (2014) and Capa et al. (2012, 2015). The holotype and paratype specimens were deposited in the Japan Agency for Marine-Earth Science and Technology (JAMSTEC), Yokosuka, Kanagawa, Japan, and the comparative material was deposited in the Coastal Branch of the Natural History Museum and Institute, Chiba (CMNH), Katsuura, Japan.

A piece of the abdomen was removed using a scalpel and placed in 1.5 ml vials containing the mixture of 9 μL of proteinase K solution (Nacalai Tesque, Kyoto, Japan) and 100 μL of 10% solution of Chelex 100 Resin (Bio-Rad, Hercules, CA). The vial was incubated at 56 °C for 30 min, followed by 100 °C for 20 min, and the resulting supernatant was used as template DNA. In addition, DNA templates were prepared for one specimen each of two sabellariid species, *Idanthyrsus okudai* Kirtley, 1994 (31 August 2015, from Tateyama, Chiba, Japan; CMNH-ZW 2341; identified according to Nishi and Kirtley 1999) and *Sabellaria isumiensis* Nishi, Bailey-Brock, Santos, Tachikawa & Kupriyanova, 2010 (16 July 2022, from Kunisaki, Oita, Japan; CMNH-ZW 2342; reported by Nishi et al. 2022b). Partial sequences of mitochondrial [16S rRNA (16S), COI] and nuclear [18S rRNA (18S), 28S rRNA (28S)] genes were obtained from the DNA templates (the holotype, the paratype, and the comparative specimens of *I. okudai* and *S. isumiensis*). The primer sets used are listed in Table 1.

**Table 1.** Primers used in the present study.

Locus	Primer	Sequence (5’–3’)^1^	Usage^2^	Reference
COI	COI1f	**GTATAAGAGACAGGACAG**AYTCNACNAAYCAYAARGAYATYGG	P	Kobayashi and Abe (2024)
	COI1r	**GTATAAGAGACAGTTCTC**GGNGGRTANACNGTYCANCC	P/S	Kobayashi and Abe (2024)
	COI2f	**GTATAAGAGACAGGACAG**CCNGAYATRKCNTTYCCNCG	P/S	Kobayashi and Abe (2024)
	COI2r	**GTATAAGAGACAGTTCTC**TAAACTTCAGGRTGNCCRAARAAYCA	P	Kobayashi and Abe (2024)
	LCO-annelid	CTCAACWAAYCAYAAAGAYATTGG	P/S	Kobayashi et al. (2022)
	HCO2198	TAAACTTCAGGGTGACCAAAAAATCA	P/S	Folmer et al. (1994)
16S	16Sa-ann	TCGMCTGTTTANCAAAAACA	P/S	Kobayashi et al. (2023)
	16Sb-ann	CGGTCTRAACTCARCTCAYG	P/S	Kobayashi et al. (2023)
	16Sann-f2	CCTGACYGTGCWAAGGTAGC	P/S	Kobayashi and Kojima (2021)
	16Sann-r2	CCYTAAGYCAACAYCGAGGT	P/S	Kobayashi and Kojima (2021)
18S	18SA1	CCTACCTGGTTGATCCTGCCAG	P	Steiner and Dreyer (2003)
	NS2	GGCTGCTGGCACCAGACTTGC	S	White et al. (1990)
	NS5	AACTTAAAGGAATTGACGGAAG	S	White et al. (1990)
	189r	TCGGAATTAACCAGACAAATC	S	Nakamura et al. (2007)
	1800r	ATGATCCTTCCGCAGGTTCACC	P	Steiner and Dreyer (2003)
	18S1f	**GTATAAGAGACAGGACAG**TGCGCTTGTCTCAAAGATTAAGCC	P	Kobayashi and Abe (2024)
	18S1r	**GTATAAGAGACAGTTCTC**GCCTGCTGCCTTCCTTRGAWGTGG	P/S	Kobayashi and Abe (2024)
	18S_2f	**GTATAAGAGACAGGACAG**ACGGGTRRCGGRGAATYAGGGTTC	P/S	Kobayashi and Abe (2024)
	18S_2r	**GTATAAGAGACAGTTCTC**GARCACTCTAATTTTTTCAAAG	P	Kobayashi and Abe (2024)
28S	D1	ACCCSCTGAAYTTAAGCAT	P/S	Brown et al. (1999)
	D3	GACGATCGATTTGCACGTCA	P/S	Vonnemann et al. (2005)
	28S_1f	**GTATAAGAGACAGGACAG**CGACCTGAGATCAGRCGRGRYTACC	P	Kobayashi and Abe (2024)
	28S_1r	**GTATAAGAGACAGTTCTC**TRCGGTMCYAYYMGTTTRMCT	P/S	Kobayashi and Abe (2024)
	28S_2f	**GTATAAGAGACAGGACAG**GAAAAGRACTTTGAAGAGAGAGT	P/S	Kobayashi and Abe (2024)
	28S_2r	**GTATAAGAGACAGTTCTC**CCTTGGTCCGTGTTTCAAGACGGGT	P	Kobayashi and Abe (2024)

^1^The regions in bold indicate binding sites used for next-generation sequencing (Kobayashi and Abe 2024), which may not be required for Sanger sequencing. ^2^P, PCR; S, sequencing.

PCR (35–40 cycles) was performed following Kobayashi and Nishi (2026) or Kobayashi et al. (2025), using the following enzymes: MightyAmp DNA Polymerase v. 3 (TaKaRa Bio, Kusatsu, Japan) for 16S; KOD One PCR Master Mix (TOYOBO, Osaka, Japan) for 18S and 28S; GoTaq G2 Hot Start Colorless Master Mix (Promega, WI, USA) for COI and 16S; or TaKaRa Ex Premier DNA Polymerase (TaKaRa Bio, Kusatsu, Japan) for COI, 18S, and 28S. The annealing temperature was 50 °C, except for COI (45–48 °C). PCR products were checked by electrophoresis on a 2% agarose gel, and the PCR products were purified using ExoSAP-IT (Thermo Fisher Scientific, Waltham, MA).

Sequencing was outsourced to Eurofins Genomics (Tokyo, Japan) or performed using an ABI 3500xl automated DNA sequencer (Applied Biosystems (ABI), MA, USA) after cycle sequencing with BigDye Terminator Cycle Sequencing Kit v3.1 (ABI) and subsequent purification by ethanol precipitation. The obtained nucleotide sequences were deposited in the DNA Data Bank of Japan (DDBJ) under DDBJ/EMBL/GenBank accession numbers (Table 2).

**Table 2.** Species used for phylogenetic analysis with GenBank accession numbers. Bold indicates sequences obtained or assembled in the present study. As only the longest sequence of each gene per species was used, sequences not used for the phylogenetic analysis are shown in parentheses.

Family	Species	COI	16S	18S	28S
Sabellariidae	*Phalacrostemma ritto* sp. nov., holotype	**(LC931932)**	**(LC931933)**	**(LC931930)**	**(LC931931)**
	*Phalacrostemma ritto* sp. nov., paratype	**LC931928**	**LC931929**	**LC931926**	**LC931927**
	*Gunnarea gaymardi*	MN045177	—	DQ317111	EU256544
	*Gesaia csiro*	MN852335	MN850402	MT524308	MT524314
	*Idanthyrsus australiensis*	KX342947	HM800975	HM800960	HM800996
	*Idanthyrsus okudai*	LC788092	**LC931936**	LC788094, **(LC931934)**	(LC788095, LC788096), **LC931935**
	*Neosabellaria cementarium*	MH242863	—	AY732223	AY732226
	*Neosabellaria upopoy*	LC855022	LC855041	LC854982	LC855002
	*Phragmatopoma caudata*	**YABB01000007**	**YABB01000001**	**YABB01000002**	**YABB01000003**
	*Phalacrostemma timoharai*	MN852334	MN850398	MT524312	MT524317
	*Phalacrostemma* sp. AM W.50676	MN852332	MN850399	MT524313	MT524318
	*Sabellaria alveolata*	KR002647	AY340479	AY340442	AY340416
	*Sabellaria isumiensis*	**LC931940, (LC931943)**	**LC931937, (LC931941)**	**LC931938, (LC931942)**	**LC931939**
Spionidae	*Marenzelleria viridis*	HQ024089	EF431973	EU418860	EU418868

A phylogenetic analysis of sabellariids was conducted using concatenated COI, 16S, 18S, and 28S gene sequences. The outgroup, *Marenzelleria viridis* (Verrill, 1873) (Spionidae), was chosen following Nishi et al. (2025). The sequences were aligned using MAFFT v. 7.294b (Katoh and Standley 2013), and ambiguously aligned regions were removed with trimAl v. 1.4.rev22 (Capella-Gutiérrez et al. 2009) using the -gappyout option, resulting in alignments of 634 (COI), 537 (16S), 1696 (18S), and 912 (28S) characters, respectively. Maximum likelihood (ML) analysis was performed in IQ-TREE v. 2.2.0.3 (Minh et al. 2020) with 1000 ultrafast bootstrap (ufBS) replicates and SH-aLRT tests (Anisimova et al. 2011). The best-fit substitution models for each locus were selected using ModelFinder (Kalyaanamoorthy et al. 2017) for the datasets as follows: GTR+F+I+G4 for COI, GTR+F+G4 for 16S, TNe+G4 for 18S, and TIM3+F+G4 for 28S. The resulting tree was edited using FigTree v. 1.4.3 (http://tree.bio.ed.ac.uk/software/figtree/).

Nucleotide sequences of 18S, 28S, and both mitochondrial genes of *Phragmatopoma caudata* (Krøyer in Mörch, 1863) included in the phylogenetic analysis were obtained from contigs assembled from NGS reads deposited in the NCBI Sequence Read Archive (SRR5818305). Assembly followed Kobayashi (2023) using SPAdes v. 4.2.0 (Bankevich et al. 2012), and gene searches were conducted with nhmmer implemented in HMMER v. 3.4 (Wheeler and Eddy 2013) and Mitos2 (Bernt et al. 2013). The assembled gene sequences were deposited as Third Party Data (TPA) in the DNA Data Bank of Japan (DDBJ) (Table 2).

## Results

### Taxonomy

**Family Sabellariidae Johnston, 1865**

#### Genus Phalacrostemma

Taxon classificationAnimaliaSabellidaSabellariidae

Marenzeller, 1895

4D5818AF-01F7-51EF-8097-4A3E96FFCDE1

##### New Japanese name.

Shinkai-Kanmurigokai.

#### Phalacrostemma ritto sp. nov.

Taxon classificationAnimaliaSabellidaSabellariidae

B5622CDC-27E3-5D46-B833-F94F454DA642

https://zoobank.org/B7D78E72-6451-4CC5-A38E-CD48516CB9A4

Figs 2, 3, 4

##### New Japanese name.

Ritto-Shinkai-Kanmurigokai.

##### Material examined.

***Holotype***: Japan • Western Mariana Ridge, Ritto Seamount, 21°47.830'N, 142°2.382'E (Fig. 1), depth 675 m; 10 October 2024, DSV*Shinkai 6500* operated from RV*Yokosuka*, Cruise YK24-15C; leg. Jimi Naoto; on a spine of a cidarid sea urchin (Fig. 2A–C); with tube; GenBank accession numbers: LC931930–LC931933 (Table 2); JAMSTEC #11820 An93-1. ***Paratype***: 1 specimen with tube broken into two pieces between the anterior region and mid-abdomen; sampling data as for the holotype; GenBank accession numbers: LC931926–LC931929 (Table 2); JAMSTEC #11820 An93-2.

##### Description.

Based on the holotype and the paratype (paratype data in parentheses).

**Figure 3.**
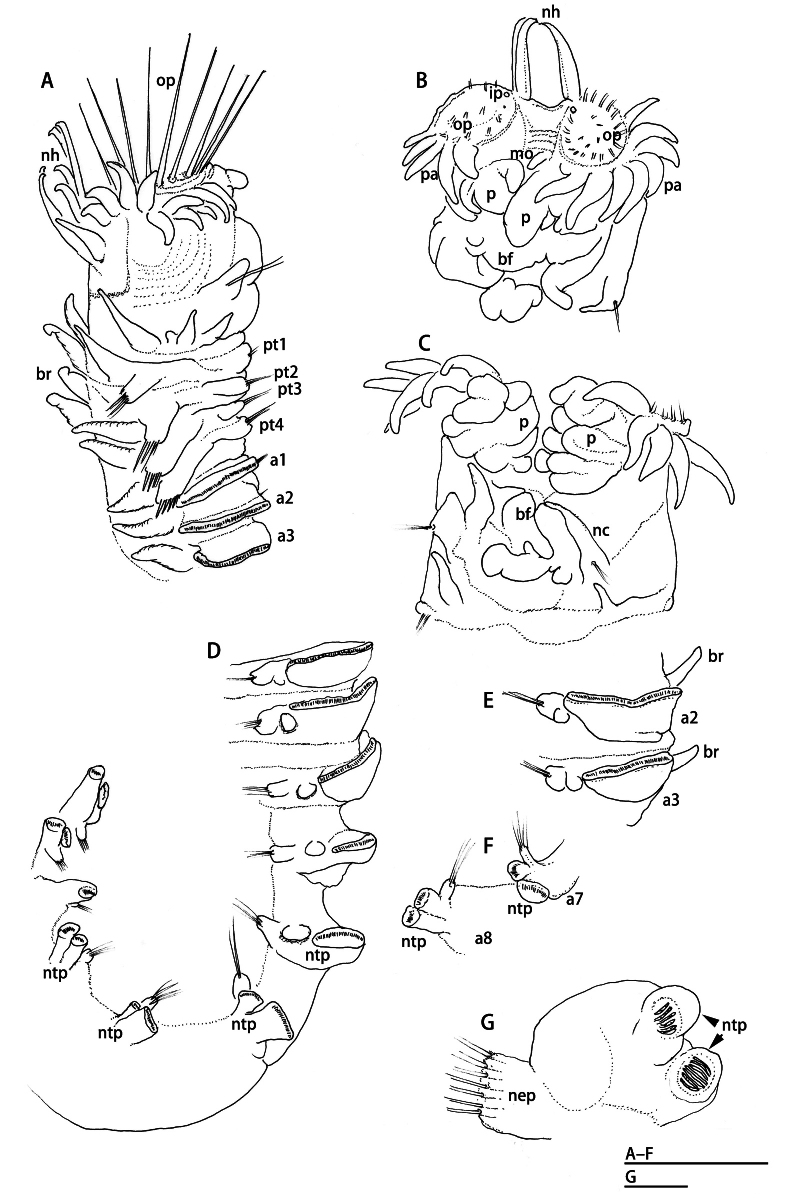
*Phalacrostemma ritto* sp. nov. **A, B, G**. Paratype; **C–F**. Holotype. **A**. Lateral view from head to parathoracic region, half of operculum omitted; **B**. Antero-ventral view of operculum, paleae omitted; **C**. Ventral view of head and operculum, paleae omitted; **D**. Ventral view of abdomen (right side), showing notopodia and neuropodia, left side omitted; **E**. Close-up view of second and third abdominal parapodia; **F**. Close-up view of 7^th^ and 8^th^ parapodia; 9^th^ parapodium also shown. pt1–4 in A indicate first to 4^th^ parathoracic chaetiger, a1–a3 in A, a2 and a3 in E, a7 and a8 in F indicate abdominal chaetiger number. Abbreviations: bf: buccal flap; br: branchia; ip: inner paleae; mo: median organ; nc: neuropodial cirri; nep: neuropodium; nh: nuchal hook; ntp: notopodium; op: opercular palea; p: palp; pa: opercular papillae; pt: parathoracx. Scale bars: 1 mm (**A–F**); 0.1 mm (**G**).

Holotype 10.0 mm in length without paleae and cauda (7.5 mm without paleae and cauda), 2.0 mm in width at parathoracic region. Operculum divided into two short, free lobes with distal ends positioned perpendicular to the longitudinal axis (Figs 2G–I, 3A–C). Opercular paleae longer than the operculum (Figs 2I, 3A); outer row with 25 paleae on the right, 28 on the left (22 on the left, 30 on the right), arranged spirally (Figs 2F–I, 3B).

**Figure 4.**
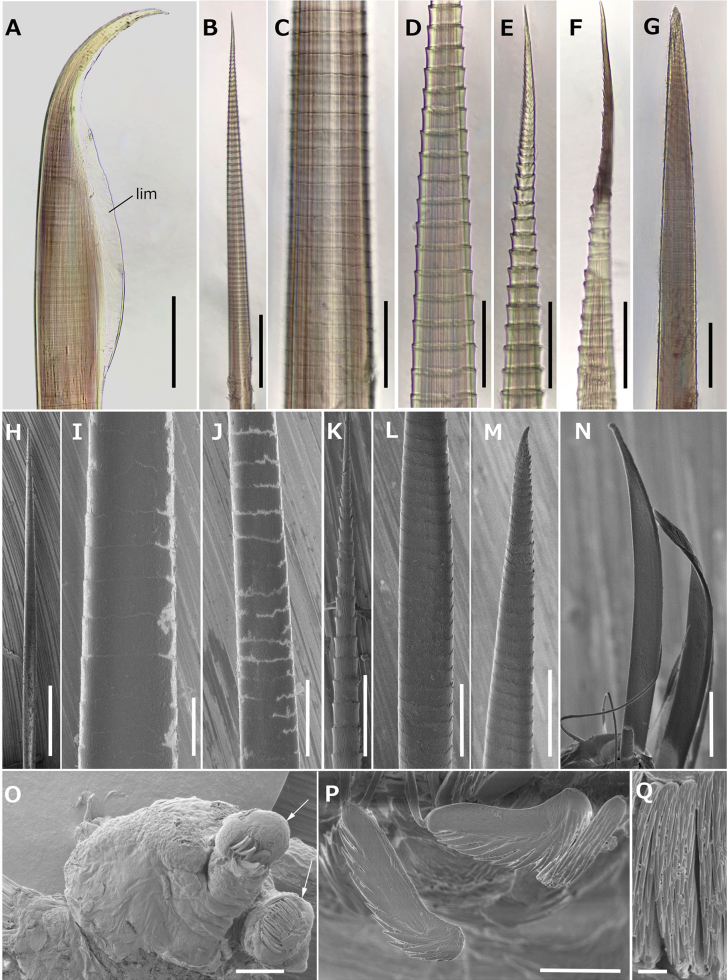
*Phalacrostemma ritto* sp. nov. **A–G**. Light photographs; **H–Q**. SEM micrographs. **A**. Nuchal hook, lim indicates limbation; **B**. Outer palea, whole view; **C**. Outer palea, basal part; **D**. Outer palea, middle part; **E**. Outer palea, tip; **F**. Outer palea, black tip; **G**. Inner palea; **H**. Outer palea, whole view; **I**. Outer palea, basal part; **J**. Outer palea, middle part; **K**. Outer palea, tip; **L**. Inner palea, middle part; **M**. Inner palea, distal tip. **N**. Parathoracic chaetiger showing lanceolate chaetae and fine capillary chaetae; **O**. Middle abdomen; showing bilobed notopodia (right) and neurochaetal tuft (left); arrows indicate lobes of notopodia. **P**. Abdominal uncini, lateral view; **Q**. Abdominal uncini, ventral view. Scale bars: 200 µm (**A, B, H**); 50 μm (**C–G, O, J–M**); 20 μm (**I, N**); 10 μm (**P**); 5 μm (**Q**).

Paleae simple, straight, with acute tips (Fig. 4B, H); distal portions of some paleae black (Figs 2I, 4E, 4F). Paleal thecae compact, with straight margins (Fig. 4B–F, H–K). Paleae arranged in two rows; inner row with one palea on the left and two on the right (two on the left and one on the right), simple and straight with tapering tips, shorter than those of the outer row (Fig. 4G, L, M). Eight conical opercular papillae on the right side and 11 on the left (13 on the right, 11 on the left), arranged peripherally to the outer paleae (Figs 2F–I, 3A–C); papillae with blunt tips (Fig. 3A–C), approximately ½ length of the outer paleae, with a basal width/length ratio of 0.6–0.8.

Five pairs of flattened nuchal hooks; concave side limbate, with tips curved at approximately 45°, wider than limbation (Figs 2H, 2I, 4A). Median organ present at the dorsal junction of opercular lobes (Figs 2H, 3B); median organ slender, about ½ length of the conical opercular papillae (Fig. 3B). Eyes not observed. Tentacular filaments absent. Buccal flaps present below a pair of palps (Figs 2G, 2I, 3B, 3C).

Chaetiger 1 (= segment 1) with neuropodial lobes and capillary neurochaetae (Fig. 3A). Chaetiger 2 with a pair of triangular lateral lobes, a pair of neuropodial lobes, and branchiae (Figs 2F, 2G, 3A). Dorsal tapering branchiae present from chaetiger 2 onwards (Figs 2H, 3A). Chaetigers 3–6 (parathorax) with two types of notochaetae—lanceolate ones interspersed with fine capillaries (Figs 3A, 4N)—and neurochaetae comprising slender lanceolate chaetae and fine capillaries.

Eleven abdominal chaetigers in the holotype and 13 in the paratype (Fig. 3D). Eight pairs of dorsal branchiae, continuing from the second thoracic chaetiger to the third abdominal chaetiger (Fig. 3A, E). Abdominal notopodia of chaetigers 1–5 unilobed, each with a single horizontal row of uncini (Fig. 3D, E); notopodia of chaetigers 6 to last bilobed, forming erect, expanded tori bearing uncini (Figs 2J, 4O). Each uncinus with three vertical rows of teeth and 8–10 columns (Fig. 4P, Q). Neuropodia with fine capillaries (Figs 2J, 3E, 3F).

Cauda long, approximately 4.0 mm in length, equal to the combined length of the abdomen and thorax in live specimens (Fig. 2D, E), smooth; about ¼ length of the abdomen in preserved specimens (Fig. 2F).

Tubes attached to sea urchin spines and composed of small sand particles (Fig. 2B, C); tubes with dark-brown inner lining; inner diameter of tubes 1.2–1.3 mm, outer diameter 2.5–3.5 mm.

##### Type locality.

Ritto Seamount, West Mariana Ridge, Japan (Fig. 1).

##### Etymology.

The specific name *ritto*, a noun in apposition, refers to the type locality, Ritto Seamount.

### Taxonomic remarks

*Phalacrostemma ritto* sp. nov. is characterized by a unique combination of morphological features: outer paleae with straight edges and without expanded thecae; five pairs of nuchal spines; absence of tentacular filaments; presence of buccal flaps; and bilobed notopodia in the middle to posterior abdomen, with each lobe bearing a row of abdominal uncini with three vertical rows of teeth. Moreover, *Phalacrostemma ritto* sp. nov. is the only species in the genus—and, to our knowledge, in the family—with bilobed notopodia in the abdomen; all other species possess unilobed abdominal notopodia.

Regarding the taxonomic value of bilobed (or unilobed) abdominal notopodia, many previous taxonomic studies lack sufficiently detailed descriptions and illustrations of the abdominal parapodia, making direct comparisons difficult. However, some published illustrations clearly show unilobed rather than bilobed parapodia (e.g. Zhang et al. 2020). In addition, the parapodia were likely examined during the extraction of the uncini for microscopic observation. If, as in our new species, the parapodia were divided into two lobes and the row of uncini were correspondingly divided, this feature would likely have been noted in the original descriptions. Thus, although this remains speculative, it is likely that sabellariid species other than the new species described here from the West Mariana Ridge possess unilobed abdominal parapodia and, consequently, a single uninterrupted row of uncini.

### Molecular results

The COI gene sequences obtained from the holotype and the paratype (658 bp) were almost identical, differing by 0.46% (*p*-distance). A BLAST search indicated that the COI sequences of the new species were significantly different from those of sabellariids available in GenBank, with the top hit being *Scolelepis mesnili* (Bellan & Lagardère, 1971) (Spionidae) (MN215916; 81% identity).

Although only the posterior part (143 bp) of the target 16S sequence (474 bp) was recovered from the holotype, the overlapping 143-bp region was identical between the holotype and the paratype. A BLAST search of the 16S sequence of the paratype returned *Phalacrostemma* sp. as the top hit (MN850399; 89.3% identity) and *P. timoharai* as the second and third hits (MN850397 and MN850398; 88.8% identity). The 18S and 28S sequences were also nearly identical between the two specimens and showed high similarity to *Phalacrostemma* sp. AM_W50676 (98.3%) and *P. timoharai* (99.3%).

### Phylogenetic analysis

**Figure 5.**
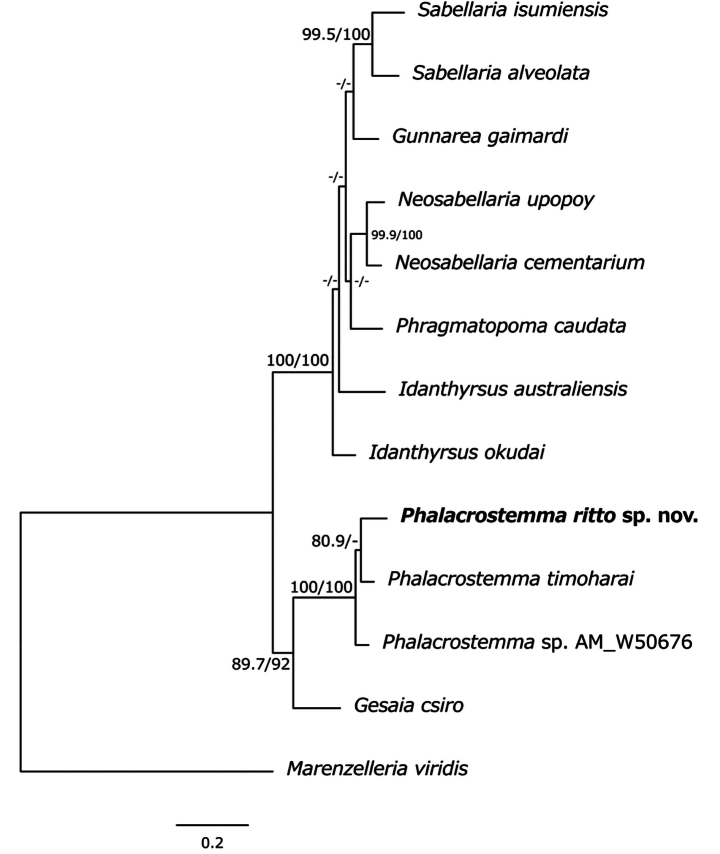
Maximum-likelihood analysis based on COI, 16S, 18S, and 28S gene sequences. Numbers above branches indicate SH-aRLT (≥80) and ultrafast bootstrap support values (≥90).

Two major clades were recovered: (1) *Gesaia csiro* + *Phalacrostemma* spp. (SH-aLRT = 89.7, ufBS = 92), and (2) a clade comprising other sabellariids (*Idanthyrsus*, *Phragmatopoma*, *Neosabellaria*, *Gunnarea*, and *Sabellaria*; SH-aLRT = 100, ufBS = 100) (Fig. 5).

Monophyly of species of *Phalacrostemma* included in the analysis—the new species, *P. timoharai*, and *Phalacrostemma* spp.—was fully supported. Two major clades were recovered: (1) *Gesaia csiro* + *Phalacrostemma* spp. (SH-aLRT = 89.7, ufBS = 92, and (2) a clade comprising other sabellariids (*Idanthyrsus*, *Phragmatopoma*, *Neosabellaria*, *Gunnarea*, and *Sabellaria*; SH-aLRT = 100, ufBS = 100) (Fig. 5). Support values for relationships among genera outside these two major clades were generally low. In the *Phalacrostemma* clade, *Phalacrostemma* sp. AM_W50676 was recovered as sister to a poorly supported clade (SH-aLRT = 80.9, ufBS = 85) comprising the new species and *P. timoharai*. All genera represented by multiple species, except for *Idanthyrsus*, were recovered as monophyletic. The phylogenetic positions of *I. okudai* and *I. australiensis* remain uncertain due to low support values.

## Discussion

*Phalacrostemma ritto* sp. nov. is the 16^th^ species of the genus, and the seventh species recorded from the Pacific following *P. setosa* (Treadwell, 1906) from off Hawaii; *P. tenue* (Augener, 1906) from off New Caledonia; *P. abyssalis* (Caullery, 1944) from off Celebes, Indonesia; *P. profundum* Lechapt & Kirtley, 1998 from off New Caledonia; *P. maloga* Hutchings, Capa & Peart, 2012, from off New South West, Australia; *P. timoharai* Zhang, Hutchings, Burghardt & Kupriyanova, 2020, from the eastern Australian abyssal zone.

*Phalacrostemma ritto* sp. nov. differs from its congeners in several diagnostic characters. *Phalacrostemma setosa* possesses tentacular filaments and lacks buccal flaps, whereas *P. ritto* sp. nov. has buccal flaps and no tentacular filaments. *Phalacrostemma tenue* has only two pairs of nuchal spines and four pairs of tentacular filaments; in contrast, *P. ritto* has five pairs of nuchal spines and lacks tentacular filaments. *Phalacrostemma abyssalis* bears nuchal spines with inflated tips, whereas those of *P. ritto* sp. nov. have curved tips. In *P. profundum*, the outer paleae have expanded thecae with irregular margins, whereas in *P. ritto* sp. nov. the thecal margins are not expanded and nearly straight. *Phalacrostemma maloga* has tentacular filaments and nuchal spines with elongate narrow tips, whereas *P. ritto* sp. nov. lacks tentacular filaments and has nuchal spines with curved tips. Finally, *P. timoharai* possesses two pairs of neuropodial cirri on chaetiger 1, whereas *P. ritto* sp. nov. has only a single pair.

Phylogenetic relationships within Sabellariidae remain insufficiently resolved (Capa and Hutchings 2014). The present analysis supports the monophyly of the *Gesaia* + *Phalacrostemma* clade, whereas relationships among the remaining genera remain unresolved. These results are consistent with those of previous molecular phylogenetic analyses (Zhang et al. 2020; Nishi et al. 2025). The closely related genera *Gesaia* and *Phalacrostemma* are morphologically very similar and differ primarily in the limbation on the nuchal hooks: in *Gesaia*, the concave margin lacks limbation, whereas in *Phalacrostemma*, the limbation is present on the concave margin (Capa and Hutchings 2014). A more comprehensive molecular phylogenetic analysis with broader taxon sampling is needed to resolve phylogenetic relationships within Sabellariidae.

## Supplementary Material

XML Treatment for Phalacrostemma

XML Treatment for Phalacrostemma ritto

## Additional information

### Artificial Intelligence (AI) use

The authors accept full responsibility for the content of the manuscript, including the disclosure of any use of AI.

No AI tools were used in the preparation of this manuscript.

### Author ORCIDs

G. Kobayashi https://orcid.org/0000-0002-5938-0824

N. Jimi https://orcid.org/0000-0001-8586-3320

Y. Fujiwara https://orcid.org/0000-0002-1833-1866

E.K. Kupriyanova https://orcid.org/0000-0003-0336-4718

### Data availability

All of the data that support the findings of this study are available in the main text.

## References

[B1] Anisimova M, Gil M, Dufayard JF, Dessimoz C, Gascuel O (2011) Survey of branch support methods demonstrates accuracy, power, and robustness of fast likelihood-based approximation schemes. Systematic Biology 60: 685–699. 10.1093/sysbio/syr041 PMC3158332 21540409

[B2] Bankevich A, Nurk S, Antipov D, Gurevich AA, Dvorkin M, Kulikov AS, Lesin VM, Nikolenko SI, Pham S, Prjibelski AD, Pyshkin AV, Sirotkin AV, Vyahhi N, Tesler G, Alekseyev MA, Pevzner PA (2012) SPAdes: a new genome assembly algorithm and its applications to single-cell sequencing. Journal of Computational Biology 19: 455–477. 10.1089/cmb.2012.0021 PMC3342519 22506599

[B3] Bernt M, Donath A, Jühling F, Externbrink F, Florentz C, Fritzsch G, Pütz J, Middendorf M, Stadler PF (2013) MITOS: improved de novo metazoan mitochondrial genome annotation. Molecular Phylogenetics and Evolution 69: 313–319. 10.1016/j.ympev.2012.08.023 22982435

[B4] Brown S, Rouse G, Hutchings P, Colgan D (1999) Assessing the usefulness of histone H3, U2 snRNA and 28S rDNA in analyses of polychaete relationships. Australian Journal of Zoology 47: 499–516. 10.1071/ZO99026

[B5] Capa M, Hutchings P (2014) Sabellariidae Johnston, 1865. In: Westheide W, Purschke W (Eds) Handbook of Zoology Online. Annelida: Polychaetes. De Gruyter, Berlin, 1–16.

[B6] Capa M, Hutchings M, Peart R (2012) Systematic revision of Sabellariidae (Polychaeta) and their relationships with other polychaetes using morphological and DNA sequence data. Zoological Journal of the Linnean Society 164: 245–284. 10.1111/j.1096-3642.2011.00767.x

[B7] Capa M, Faroni-Perez L, Hutchings P (2015) Sabellariidae from Lizard Island, Great Barrier Reef, including a new species of *Lygdamis* and notes on external morphology of the median organ. Zootaxa 4019(1): 184–206. 10.11646/zootaxa.4019.1.10 26624070

[B8] Capella-Gutiérrez S, Silla-Martínez JM, Gabaldón T (2009) TrimAl: a tool for automated alignment trimming in large-scale phylogenetic analyses. Bioinformatics 25: 1972–1973. 10.1093/bioinformatics/btp348 PMC2712344 19505945

[B9] Chávez-López Y (2022) New species of sabellariids (Annelida: Sabellariidae) from the Caribbean Sea and the Gulf of Mexico. European Journal of Taxonomy 831: 109–148. 10.5852/ejt.2022.831.1873

[B10] Folmer O, Black M, Hoeh W, Lutz R, Vrijenhoek R (1994) DNA primers for amplification of mitochondrial cytochrome c oxidase subunit I from diverse metazoan invertebrates. Molecular Marine Biology and Biotechnology 3: 294–299. 10.1371/journal.pone.0013102 7881515

[B11] Hutchings P, Capa M, Peart R (2012) Revision of the Australian Sabellariidae (Polychaeta) and description of eight new species. Zootaxa 3306(1): 1–60. 10.11646/zootaxa.3306.1.1

[B12] Imajima M (2001) Deep-sea benthic polychaetous annelids of Tosa Bay, southwestern Japan. In: Fujita T, Saito T, Takeda M (Eds) Deep-Sea Fauna and Pollutants in Tosa Bay. National Science Museum Monographs No. 20. National Museum of Nature and Science, Tokyo, 31–100.

[B13] Imajima M (2006) Polychaetous annelids from Sagami Bay and the Sagami Sea, central Japan. Memoirs of National Science Museum, Tokyo (40): 317–407.

[B14] Imajima M (2009) Deep-sea benthic polychaetes off Pacific coast of the northern Honshu, Japan. National Museum of Nature and Science Monographs 39: 39–192.

[B15] Jimi N (2024) The polychaetous annelids of Japan: updated checklist of known species. Species Diversity 29: 337–377. 10.12782/specdiv.29.337

[B16] Kalyaanamoorthy S, Minh BQ, Wong TKF, von Haeseler A, Jermiin LS (2017) ModelFinder: fast model selection for accurate phylogenetic estimates. Nature Methods 14: 587–589. 10.1038/nmeth.4285 PMC5453245 28481363

[B17] Katoh K, Standley DM (2013) MAFFT multiple sequence alignment software version 7: improvements in performance and usability. Molecular Biology and Evolution 30: 772–780. 10.1093/molbev/mst010 PMC3603318 23329690

[B18] Kirtley DW (1994) A Review and Taxonomic Revision of the Family Sabellariidae Johnston, 1865 (Annelida: Polychaeta). Sabecon Press Science Series, Vero Beach, Florida, 223 pp.

[B19] Kobayashi G, Kojima S (2021) *Travisia sanrikuensis*, a new species of Travisiidae (Annelida) from the lower bathyal zone of the northwestern Pacific. Species Diversity 26: 131–136. 10.12782/specdiv.26.131

[B20] Kobayashi G, Itoh H, Kojima S (2022) Mitogenome of a stink worm (Annelida: Travisiidae) includes degenerate group II intron that is also found in five congeneric species. Scientific Reports 12: 4449. 10.1038/s41598-022-08103-5 PMC8924214 35292662

[B21] Kobayashi G (2023) Buried treasure in a public repository: mining mitochondrial genes of 32 annelid species from sequence reads deposited in the Sequence Read Archive (SRA). PeerJ 11: e16446. 10.7717/peerj.16446 PMC10693233 38047014

[B22] Kobayashi G, Itoh H, Nakajima N (2023) First report of the mitogenome of the invasive reef-building polychaete *Ficopomatus enigmaticus* (Annelida: Serpulidae) and a cryptic lineage from the Japanese Archipelago. Molecular Biology Reports 50: 7183–7196. 10.1007/s11033-023-08647-3 37407804

[B23] Kobayashi G, Abe H (2024) Cost-efficient PCR based DNA barcoding of marine invertebrate specimens with NovaSeq amplicon sequencing. Molecular Biology Reports 51: 887. 10.1007/s11033-024-09811-z 39105821

[B24] Kobayashi G, Nishi E (2026) Becoming worldwide: first record of the invasive fan worm *Branchiomma luctuosum* (Annelida: Sabellidae) in the Pacific, based on a specimen from Japan. Plankton and Benthos Research 21(1): 7–15. 10.3800/pbr.21.7

[B25] Kobayashi G, Sekiguchi S, Fukumori H (2025) Predation by a tiny phoxichilidiid sea spider on a juvenile terebellid polychaete *Thelepus japonicus*. Marine Biology Research 21(6–7): 271–275. 10.1080/17451000.2025.2512433

[B26] Koeda K, Sado T, Hata H, Fujiwara Y (2024) Redescription and first Japanese seamount record of *Stethopristes eos* (Zeiformes; Parazenidae). Zootaxa 5399(5): 579–586. 10.11646/zootaxa.5399.5.7 38480118

[B27] Komai T, Tsuchida S, Fujiwara Y (2022) New record of a rarely collected caridean shrimp *Bathypalaemonella pandaloides* (Rathbun, 1906) (Decapoda: Bathypalaemonellidae) from the West Mariana Ridge, northwestern Pacific. Zootaxa 5129(2): 272–284. 10.11646/zootaxa.5129.2.7 36101136

[B28] Komai T, Tsuchida S, Fujiwara Y (2023a) Squat lobsters of the superfamily Chirostyloidea (Decapoda: Anomura) from seamounts on the Nishi-Shichito and Mariana ridges, North-West Pacific off Japan, with descriptions of two new species. Zootaxa 5293(1): 45–73. 10.11646/zootaxa.5293.1.2 37518497

[B29] Komai T, Tsuchida S, Fujiwara Y (2023b) A new deep-sea palaemonid shrimp assigned to *Periclimenes* Costa, 1844 (Decapoda: Caridea) from the West Mariana Ridge, northwestern Pacific. Zootaxa 5231(4): 376–392. 10.11646/zootaxa.5231.4.2 37045138

[B30] Lechapt JP, Kirtley DW (1998) New species of bathyal and abyssal Sabellariidae (Annelida: Polychaeta) from near New Caledonia (southwest Pacific Ocean). Proceedings of the Biological Society of Washington 111: 807–822.

[B31] Minh BQ, Schmidt HA, Chernomor O, Schrempf D, Woodhams MD, von Haeseler A, Lanfear R (2020) IQ-TREE 2: new models and efficient methods for phylogenetic inference in the genomic era. Molecular Biology and Evolution 37: 1530–1534. 10.1093/molbev/msaa015 PMC7182206 32011700

[B32] Ministry of the Environment (2020a) Mid-Ocean Ridge/West Mariana Ridge Northern Offshore Seafloor Natural Environment Conservation Area Designation and Conservation Plan. Ministry of the Environment, Tokyo, 8 pp. [In Japanese]

[B33] Ministry of the Environment (2020b) West Seven Island Ridge Offshore Submarine Natural Environment Conservation Area Designation and Conservation Plan. Ministry of the Environment, Tokyo, 8 pp. [In Japanese]

[B34] Nakamura K, Kano Y, Suzuki N, Namatame T, Kosaku A (2007) 18S rRNA phylogeny of sea spiders with emphasis on the position of Rhynchothoracidae. Marine Biology 153: 213–223. 10.1007/s00227-007-0803-0

[B35] Nation JL (1983) A new method using hexamethyldisilazane for preparation of soft insect tissues for scanning electron microscopy. Stain Technology 58(6): 347–351. 10.3109/10520298309066811 6679126

[B36] Nishi E, Kirtley DW (1999) Three new species of Sabellariidae (Polychaeta) from Japan. Natural History Research 5(2): 93–105. https://www.chiba-muse.or.jp/NATURAL/publication/nhr_5-2_6nishi.pdf

[B37] Nishi E, Kato T (2002) Sabellariid polychaetes from Japan. Taxa, Proceedings of the Japanese Society of Systematic Zoology 13: 5–17. [In Japanese]

[B38] Nishi E, Bailey-Brock JH, Santos ASD, Tachikawa H, Kupriyanova EK (2010) *Sabellaria isumiensis* n. sp. (Annelida: Polychaeta: Sabellariidae) from shallow waters off Onjuku, Boso Peninsula, Japan, and re-descriptions of three Indo-West Pacific sabellariid species. Zootaxa 2680(1): 1–25. 10.11646/zootaxa.2680.1.1

[B39] Nishi E, Abe H, Tanaka K, Jimi N, Kupriyanova EK (2022a) A new species of the *Spirobranchus kraussii* complex, *S. akitsushima* (Annelida, Polychaeta, Serpulidae), from the rocky intertidal zone of Japan. ZooKeys 1100: 1–28. 10.3897/zookeys.1100.79569 PMC9848934 36760394

[B40] Nishi E, Abe H, Makiguchi N, Aoki M, Ueno R, Kitanishi S, Hamaguchi M (2022b) Record of *Sabellaria isumiensis* (Annelida: polychaete: Sabellariidae) from the intertidal rocky shore of Kurotsuzaki, Kunisaki, Oita Prefecture. Nanki Seibutsu 64(2): 140–142. [In Japanese]

[B41] Nishi E, Abe H, Jimi N, Tanaka K, Kobayashi G, Makiguchi N, Kupriyanova EK (2025) Description of a reef-forming *Neosabellaria upopoy* sp. nov. (Annelida: Polychaeta: Sabellariidae) from shallow waters off Iburi, Hokkaido, Japan, with notes on its reproduction and early development. Sessile Organisms 42(1): 1–17. 10.4282/sosj.42.1

[B42] Read G, Fauchald K [Eds] (2025a) World Polychaeta Database. Sabellariidae Johnston, 1865. World Register of Marine Species. https://www.marinespecies.org/aphia.php?p=taxdetails&id=979 [Accessed on: 20 August 2025]

[B43] Read G, Fauchald K [Eds] (2025b) World Polychaeta Database. *Phalacrostemma* Marenzeller, 1895. World Register of Marine Species. https://marinespecies.org/aphia.php?p=taxdetails&id=129519 [Accessed on: 20 August 2025]

[B44] Steiner G, Dreyer H (2003) Molecular phylogeny of Scaphopoda (Mollusca) inferred from 18S rDNA sequences: Support for a Scaphopoda–Cephalopoda clade. Zoologica Scripta 32: 343–356. 10.1046/j.1463-6409.2003.00121.x

[B45] Vonnemann V, Schrödl M, Klussmann-Kolb A, Wägele H (2005) Reconstruction of the phylogeny of the Opisthobranchia (Mollusca: Gastropoda) by means of 18S and 28S rRNA gene sequences. Journal of Molluscan Studies 71: 113–125. 10.1093/mollus/eyi014

[B46] Wheeler TJ, Eddy SR (2013) nhmmer: DNA homology search with profile HMMs. Bioinformatics 29: 2487–2489. 10.1093/bioinformatics/btt403 PMC3777106 23842809

[B47] White TJ, Bruns T, Lee S, Taylor JW (1990) Amplification and direct sequencing of fungal ribosomal RNA genes for phylogenetics. In: Innis MA, Gelfand DH, Sninsky JJ, White TJ (Eds) PCR Protocols: A Guide to Methods and Applications. Academic Press, Cambridge, MA, 315–322. 10.1016/b978-0-12-372180-8.50042-1

[B48] Zhang J, Hutchings P, Burghardt I, Kupriyanova EK (2020) Two new species of Sabellariidae (Annelida, Polychaeta) from the abyss of eastern Australia. Zootaxa 4821(3): 487–510. 10.11646/zootaxa.4821.3.4 33056312

